# Parental migration and smoking behavior of left-behind children: evidence from a survey in rural Anhui, China

**DOI:** 10.1186/s12939-016-0416-7

**Published:** 2016-08-05

**Authors:** Tingting Yang, Cuicui Li, Chengchao Zhou, Shan Jiang, Jie Chu, Alexis Medina, Scott Rozelle

**Affiliations:** 1 School of Public Health, Shandong University, 44 Wen-hua-xi Road, Jinan, Shandong 250012 China; 2 Shandong Centre for Disease Control and Prevention, Jinan, 250014 China; 3 Freeman Spogli Institute, Stanford University, Stanford, CA 94305 USA

**Keywords:** Parental migration, Current smoking, Left-behind children, Risk factors, Cross-sectional study

## Abstract

**Background:**

Parental migration is most an important factor affecting children’s behaviors. Few studies have addressed the association between parental migration and children’s smoking behavior in China. This study aims to estimate the current smoking prevalence among children, evaluate the association of parental migration and the smoking behavior of children and identify factors associated with smoking behavior among left-behind children (LBC).

**Methods:**

A cross-sectional study was conducted in 6 cities in Anhui province during July and August, 2012. All participants were interviewed face-to-face using a standardized questionnaire. Only children 10 to 14 years old that live in rural villages for at least 6 months during the previous year were included in the study.

**Results:**

A total of 1343 children met the sampling criteria and participated in the study. Of these, 56 % are LBC and 44 % live with both parents. The average rate of smoking is 3.4 %. The rate of smoking is statistically higher for LBC with both parents out (rate = 6.1 %; **OR** = 5.59, ***P*** < 0.001) than for children living with both parents (1.4 %). Similarly, the rate of LBC with father home only (rate = 5.0 %; **OR** = 5.60, ***P*** = 0.005) is also statistically higher than for children living with both parents when controlling other variables. Factors affecting the smoking behavior of LBC, include gender (i.e., boys), (perceived) school performance and primary caregiver.

**Conclusions:**

Parental migration is associated with a significant increase in smoking behavior among children. Intervention studies that target LBC would help to develop strategies to reduce smoking among rural children. Gender-specific strategies and anti-smoking education also appears to be needed to reduce tobacco use among rural LBC.

## Background

Tobacco use, in particular smoking, is one of the major preventable causes of morbidity and mortality globally. The World Health Organization (WHO) attributes 6 million deaths to tobacco each year. Nearly 70 % of these deaths occur in low- and middle-income counties. If the current trend continues, the annual death rate will rise to more than 8 million people per year. Eighty percent of these deaths are projected to occur in low- and middle-income countries by 2030 [1, 2].

Tobacco use is among the most serious public health problems in China [3, 4]. Policy makers and health officials are increasingly concerned about the smoking behavior of adolescents and young adults, as these individuals are said to particularly susceptible to initiating tobacco use [5–8]. Some studies indicated that most smoking behavior in China begins between the ages of 10 to 15 years; a quarter of individuals that are smoking today began to use tobacco before the age of 10 [9]. Adolescents often underestimate the risks of smoking behavior and the likelihood of becoming addicted to tobacco use. The younger children are when they first start using tobacco, the more likely they are to become regular users and the less likely they are to quit [10, 11]. Health officials believe that preventing children from being stricken by tobacco use may be able to reduce tobacco-related morbidity and mortality in the future.

Many studies suggest that the family environment is one of the most critical determinants of adolescent health behavior and development [12, 13]. In that case, parental migration may play an important role in the smoking behavior of children due to lack of monitoring. In China, as society undergoes rapid urbanization and economic change, a large number of rural residents have migrated to China’s major cities to seek employment opportunities. In many cases they have chosen to leave their children behind (called left behind children, LBC) in rural areas rather than bring them to the city where services such as education can be difficult to access [14, 15]. LBC refers to children who stay at home in their rural village/community when either one or both of their parents migrate elsewhere to work for at least six months during the previous year [16]. A report released in May 2013 by the All-China Women’s Federation estimated that over 61 million children were left in their rural homes by one or both of their parents migrating for work. This number of children accounts for about 37.7 % of rural children [17]. Two studies conducted in China indicated that parental migration was a risk factor for unhealthy behaviors among children in rural China [18, 19]. Another study also found that adolescents whose mothers migrated from home to work elsewhere were at elevated risk for smoking [20]. However, there is also a dearth of studies which attempt to discern the association of parental migration with smoking behavior of children.

There are three objectives in the present study. First, we estimate the prevalence of smoking among children living with parents and LBC. Second, we evaluate the association between parental migration and the smoking behavior of children. Finally, we identify socio-demographic factors associated with the smoking behavior of LBC. Ultimately, we hope the findings of the study will help in developing interventions that can reduce tobacco use among children.

## Methods

### Study site

**Fig. 1:**
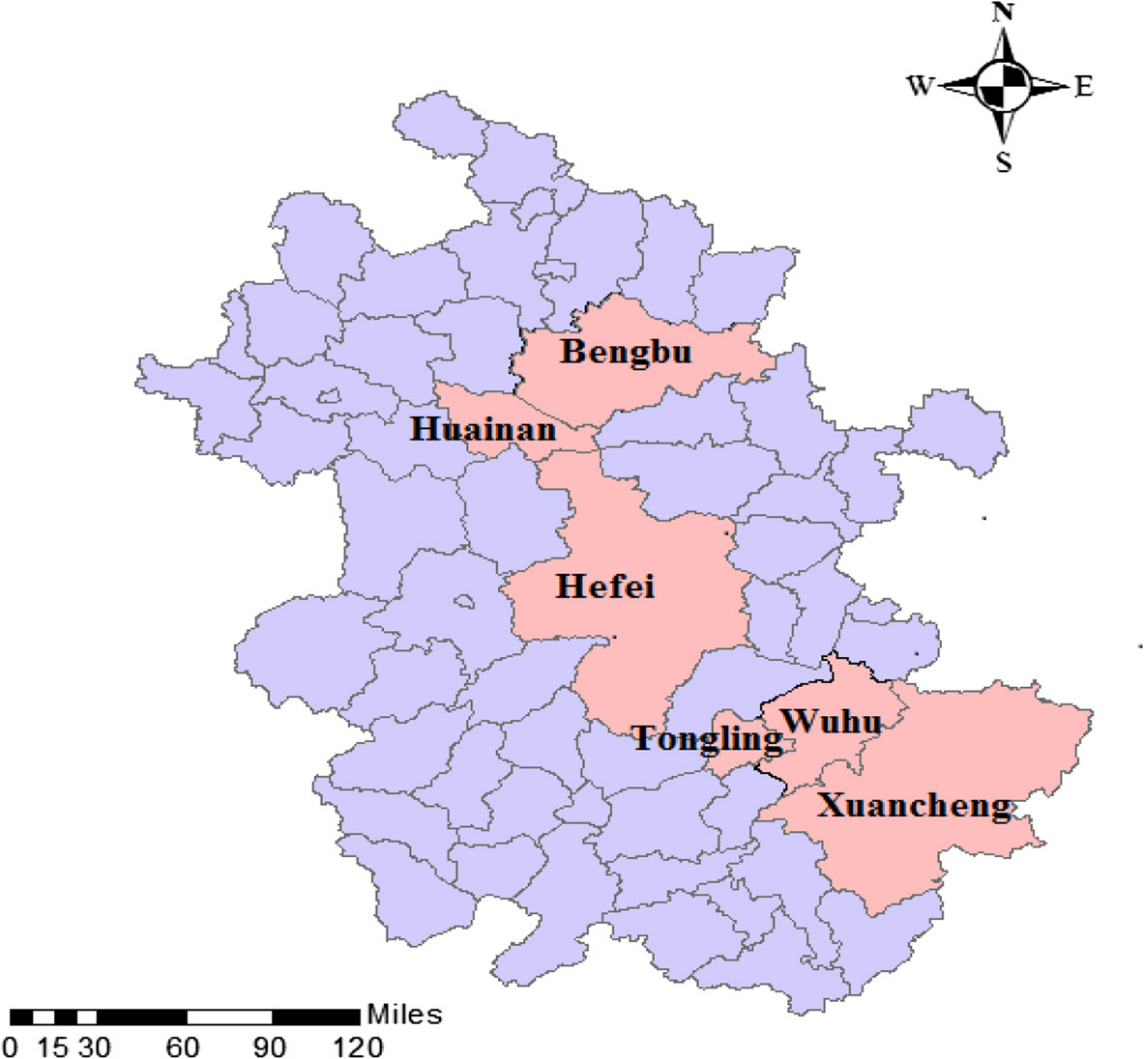
Location of the study sites in Anhui province

This study was conducted in Anhui province in central China. With a population of nearly 70 million, Anhui is divided into 16 municipalities and 105 counties [21]. A national survey indicates that Anhui ranks third in terms of the number of LBC [17]. It is estimated that there are more than 4.4 million LBC in Anhui, accounting for over 50 % of rural children in the province [17].In 2007, the prevalence of current smoking,among adults in Anhui province was 33.8 %, with a rate of 67.0 % among men and 2.6 % among women in the same province [22].A total of six cities (that is, administrative municipalities that encompass large rural areas) were randomly selected to serve as the study cities after considering the GDP level (high, middle and low) and also location (northern, central and southern) of the cities, Fig. 1 shows the location of the six study sites.

### Study participants

To recruit our sample’s children, we used a cluster random sampling approach, using townships as the basis of our cluster. Step one of the study’s sampling protocol was to stratify all prefecture-level cities into three groups according to per capita gross domestic product, and to select two cities from each group. The location (northern, central and southern regions in Anhui Province, China) was also considered when selecting sample cities. Next, we obtained a list of all the townships in each of the cities, and randomly select one township in each of the selected six cities. Third, in each township three villages with a population of 1000 residents (including all age groups) or above were randomly sampled from a list of all villages. Fourth, we used official records and telephone calls to the heads of the selected villages in order to conduct our survey. Fifth, we chose the study participants. The participants included all of those that met the following criteria: children that were ages 10 to 14 years who had lived in each sample village for at least 6 months during the previous year. A total of 1343 children were recruited for the study.

### Data collection

A survey was conducted between July and August, 2012. All participants were interviewed face-to-face using a standardized survey questionnaire. The survey instrument included personal demographic information (age, sex, grade, etc.), parental migration status, other information about the occupation of parents, and children’s health behavior. The interviewers assisted children to complete a questionnaire in their homes without the presence of caregivers. The survey was conducted by graduate students from Shandong University. Before the survey the investigators were trained rigorously on the content of the questionnaires and on proper interviewing techniques.

### Definitions and measures

LBC were categorized into three types: mothers at home but fathers out; fathers at home but mother out; and both parents gone. Smoking in this study means that the individual was known to have engaged in smoking cigarettes during one or more days at some point in the past 30 days (before the survey).In this study, a primary caregiver is the person who takes primary responsibility for caring the children and had lived together with the children for more than 6 months in the past year. Primary caregivers were categorized into four types in this study: mother, father, grandparents and other relatives. We used a question to measure “perceived school performance”: “How do you think your teacher estimates your school performance compared with other classmates?”, and the answer categories were: very good, good, average, and less than average. When analyzing the variable, we merged the “very good” and “good” into one group of “good”.

Age of the children were categorized into two types: 10–12 years (most of the children with such age were still elementary school students) and 13–14 years (most of the children with such age were junior school students). Grade of the children were also categorized into two types: grade 6 and below (elementary school students) and grade 7 and above (junior school students). We categorized “length of parental migration” into three types in this study: 1 year and below, 1 to 5 years, 6 years and above. We categorized “frequency of parental contacts” into four types: everyday, once a week, once every 1 to 4 weeks, and once a month or longer. We also classified “parental frequency of home visits” into four types: once each 3 months, once each 4 to 6 months, once each 7 to 12 months, and once each 12 months or longer.

### Data analysis

The data were double entered using EPI Data 6.04. Both copies of the data were checked for consistency. The statistical package SPSS 13.0 was used to analyze the data. Descriptive analyses were performed using frequencies, percentages, and mean ± standard deviation (SD). Chi-square tests and t-tests were performed to evaluate differences for categorical and quantitative variables, respectively.

The smoking behavior of children was compared among different subgroups using univariate logistic regression analysis. Multivariate logistic regression model was employed to analyze risk factors associated with smoking behavior and also to control possible confounding of the influences of the factors (e.g. gender, age, grade of the children, perceived school performance, frequency of parental contact, frequency of parental visits, primary caregiver).

## Results

Table 1 shows basic information about the overall sample. The sample is subdivided by parental migration status. A total of 1343 children were eligible for analysis. LBC with the age from 10 to 12 accounted for 57 %, which is higher than that among non-LBC (50 %) (*p* = 0.01). In the sample, 56 % were boys and 77 % were grade 6 and below (or in elementary school). About 56 % of the participants were LBC.

**Table 1:** Socio-demographic characteristics of study participants in rural Anhui, China, 2012

Variable	Overall	Left-behind	Non-left-behind	*P-value*
*n*	1343 (100.0 %)	757 (56.4 %)	586 (43.6 %)	
Gender				0.93
Male	750 (55.8 %)	422 (55.7 %)	328 (56.0 %)	
Female	593 (44.2 %)	335 (44.3 %)	258 (44.0 %)	
Age				0.01
10–12	723 (53.8 %)	432 (57.1 %)	291 (49.7 %)	
13–14	620 (46.2 %)	325 (42.9 %)	295 (50.3 %)	
Grade				0.00
≤ 6	1027 (76.5 %)	604 (79.8 %)	423 (72.2 %)	
≥ 7	316 (23.5 %)	153 (20.2 %)	163 (27.8 %)	
Only child				0.09
Yes	670 (49.9 %)	362 (47.8 %)	308 (52.6 %)	
No	673 (50.1 %)	395 (52.2 %)	278 (47.4 %)	
Perceived school performance				0.79
Good	632 (46.2 %)	356 (45.6 %)	276 (47.1 %)	
Moderate	532 (38.9 %)	310 (39.7 %)	222 (37.9 %)	
Bad	203 (14.9 %)	115 (14.7 %)	88 (15.0 %)	

Source: Authors’ analyses of survey data that the authors and collaborators conducted in rural Anhui province, China

Of the 757 LBC (see Table 2), those children who did not live with either parent (that is, both parents had outmigrated) accounted for over 50 %. Of the remaining LBC, 37 % lived with their mother only; 13 % lived with their father only. In our sample, in the case of only 21 % of the LBC, the migrant parent had contact (usually by phone) with the LBC every day. According to our data, the mother was the primary caregiver in 37 % of the cases. The paternal grandparents were the primary caregiver in 35 % of the cases.

**Table 2:** Characteristics (related to migration) of left-behind children (LBC) in rural Anhui, China, 2012

Variable	Number	Percentage (%)
*n*	757	100.0
Migration status		
Father out only	281	37.1
Mother out only	100	13.2
Both out	376	49.7
Length of parental migration (years)		
≤ 1	168	22.2
1–5	403	53.2
≥ 6	186	24.6
Frequency of parental contact^a^		
Everyday	160	21.1
Once a week	342	45.2
Once every 1 to 4 weeks	145	19.2
Once a month (or longer)	110	14.5
Frequency of home visits by parents		
Once each 3 months	118	15.6
Once each 4 to 6 months	287	37.9
Once each 7 to 12 months	299	39.5
Once each 12 months (or longer)	53	7.0
Primary caregiver		
Mother	281	37.1
Father	100	13.2
Grandparents	263	34.7
Other relatives	113	14.9

Source: Authors’ analyses of survey data that the authors and collaborators conducted in rural Anhui province, China

^a^The parents contacted with their children left behind usually by phone (over 95 %)

**Fig. 2:**
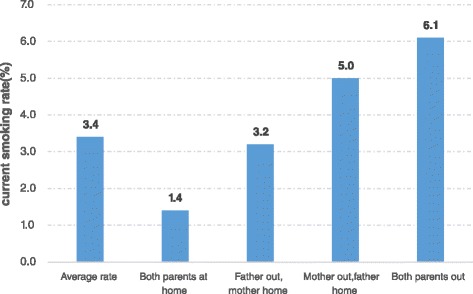
Smoking rates of children by different family types

In order to understand the correlation between the migration status of the parents and the smoking behavior of the sample children, we describe the rate of smoking by parental migration status (Fig. 2). Our data show that the average rate of smoking among the sample participants was 3.4 % (45/1343). When we examine the association of smoking behavior of children with the migration status of their parents, we find that the rate of smoking was the highest when both parents had migrated (6.1 %). In contrast, the lowest rate of smoking of children was found when both parents were at home (1.4 %). The rate of smoking was 5.0 % with the mother having migrated (and the father was at home); it was 3.2 % with the father having migrated while the mother was at home.

We present the results of the study to estimate the effect of parental migration on smoking behavior of children using two models. The unadjusted model shows that when the mother migrates and when both parents migrate, the rate of smoking is statistically significant compared to when both parents are at home (Table 3). When controlling for other variables, the smoking rates of children for households in which the mother or both parents migrate are also statistically higher than the households in which both parents are at home.

**Table 3:** Association of current smoking behavior of rural children and parental migration status, Anhui, China 2012

Variable	Model 1 (No covariates)			Model 2 (Covariates)		
	*P*	OR	OR 95 % CI	*P*	OR	OR 95 % CI
Migration status						
Both parents at home		1.0			1.0	
Father out, mother home	0.076	2.39	0.91–6.26	0.063	3.20	0.94–8.77
Mother out, father home	0.021	3.80	1.22–11.87	0.005	5.60	1.67–18.73
Both parents out	0.000	4.71	2.08–10.64	0.000	5.59	2.38–13.15
Gender						
Female					1.0	
Male				0.000	10.86	3.28–35.91
Grade						
≤ 6					1.0	
≥ 7				0.002	2.80	1.44–5.45
Only child						
Yes					1.0	
No				0.088	1.82	0.92–3.62
Perceived school performance						
Good					1.0	
Moderate				0.790	1.12	0.48–2.63
Bad				0.000	5.08	2.36–10.93
Observations	1343			1343		

Source: Authors’ analyses of survey data that the authors and collaborators conducted in rural Anhui province, China

The analysis also shows that the rate of smoking can be compared across different subgroups of LBC using univariate analysis. The analysis shows that a) those LBC who are male; b) those LBC whose ages are older; c) those LBC who are in 7^th^ grade and above; d) those LBC whose perceived performance is poor; e) those LBC whose parental migratory length of time is between 1 and 5 years; f) those LBC whose frequency of parents contact and home visits frequency are higher; and g) those LBC who are cared for by other relatives are more likely to smoke. The multi-logistic regression analysis also identifies factors associated with the smoking behavior of LBC (Table 4). According to the analysis, LBC that are male; LBC who perceive their school performance to be low; and LBC who are cared for by other relatives tend to be smokers.

**Table 4:** Factors associated with current smoking behavior among rural left-behind children (LBC), Anhui, China 2012

Variable	Smoker (%)	Non-smoker (%)	*P*	OR_c_^*^	OR_c_95%CI	*P*	OR_a_^#^	OR_a_95%CI
*n = 757*	37(4.9)	720(95.1)						
Gender						0.000		
Female (ref.)^†^	3(0.9)	332(99.1)		1.0			1.0	
Male	34(8.1)	388(91.9)	0.000	9.70	2.95–31.86	0.000	8.99	2.65–30.44
Age						0.149		
10–12 (ref.)	12(2.8)	420(97.2)		1.0			1.0	
13–14	25(7.7)	300(92.3)	0.002	2.92	1.44–5.90	0.149	1.98	0.78–5.01
Grade						0.366		
≤ 6 (ref.)	22(3.6)	582(96.4)		1.0			1.0	
≥ 7	15(9.8)	138(90.2)	0.002	2.88	1.45–5.69	0.366	1.54	0.60–3.94
Perceived school performance						0.010		
Good (ref.)	10(2.9)	338(97.1)		1.0			1.0	
Moderate	11(3.6)	291(96.4)	0.581	1.28	0.54–3.05	0.388	1.51	0.59–3.85
Bad	16(15.0)	91(85.0)	0.000	5.94	2.61–13.54	0.004	3.92	1.56–9.84
Length of parental migration (years)						0.217		
≤ 1 (ref.)	3(1.8)	165(98.2)		1.0			1.0	
1–5	27(6.7)	376(93.3)	0.026	3.95	1.18–13.20	0.094	2.99	0.83–10.75
≥ 6	7(3.8)	179(96.2)	0.273	2.15	0.55–8.46	0.322	2.88	0.49–8.91
Migration status						NA^△^		
Both parents out (ref.)	23(6.1)	353(93.9)		1.0				
Father out, mother home	9(3.2)	272(96.8)	0.091	0.51	0.23–1.12			
Mother out, father home	5(5.0)	95(95.0)	0.674	0.81	0.30–2.18			
Frequency of parental contact^※^						0.573		
Everyday (ref.)	4(2.5)	156(97.5)					1.0	
Once a week	13(3.8)	329(96.2)	0.456	1.54	0.49–4.80	0.775	0.83	0.24–2.93
Once every 1 to 4 weeks	9(6.2)	136(93.8)	0.121	2.58	0.78–8.57	0.797	1.20	0.31–4.61
Once a month (or longer)	11(10.0)	99(90.0)	0.014	4.33	1.34–13.99	0.477	1.62	0.43–6.06
Parental frequency of home visits						0.338		
Once each 3 months (ref.)	3(2.5)	115(97.5)					1.0	
Once each 4 to 6 months	7(2.4)	280(97.6)	0.951	0.96	0.24–3.77	0.804	1.21	0.27–5.46
Once each 7 to 12 months	22(7.4)	277(92.6)	0.075	3.05	0.89–10.37	0.212	2.46	0.60–10.04
Once each 12 months (or longer)	5(9.4)	48(90.6)	0.045	3.99	1.12–17.37	0.231	2.71	0.53–13.81
Primary caregiver						0.079		
Other relatives (ref.)	13(11.5)	100(88.5)					1.0	
Mother	9(3.2)	272(96.8)	0.002	0.26	0.11–0.61	0.031	0.35	0.13–0.91
Father	5(5.0)	95(95.0)	0.097	0.41	0.14–1.18	0.309	0.54	0.16–1.77
Grandparents	10(3.8)	253(96.2)	0.006	0.30	0.13–0.72	0.024	0.33	0.13–0.87

Source: Authors’ analyses of survey data that the authors and collaborators conducted in rural Anhui province, China. Note: The p-value for the model is 0.000

^*^ OR_c_:crude odds ratio;^#^ OR_a_: adjusted odds ratio

^†^ ref.: reference group

^△^NA: not applicable; ^※^The parents contacted with their children left behind usually by phone (over 95 %)

## Discussion

This study shows that the current smoking prevalence among the participants is 3.4 %. The rate of smoking among LBC is higher than that among non-LBC. A gender difference is observed among LBC in this study, those who are male are more likely to be current smokers. Perceived school performance is also found to be associated with smoking. Interestingly, we also find that the type of caregiver is associated with smoking among the LBC.

Many previous studies indicate that parental migration affects the physical and mental health of their children when they are left behind in their home communities [23, 24]. However, research that addresses the relationship between parental migration and specific health behavior of children is scarce. The current study was conducted as a way to identify and compare the prevalence of smoking between rural children living with both of parents and LBC. Our study demonstrates that an average of 3.4 % of all participants were current smokers. The rate of smoking in the current study is lower than that in Chongqing (6.3 %), Tianjin (5.7 %) and Guangdong (4.5 %), according to researchers that conducted the 1999 Global Youth Tobacco Survey (GYTS—a study with participants ages 13 to 15). The rate of smoking in this study is higher than that in Shandong (2.4 %--also according to GYTS) as well as according to a recent survey of Shandong (1.2 %) in 2012 [25, 26]. The variations in the rates of prevalence could be explained by the age differences of the participants in the sample or by cultural discrepancies in the different study areas or by the way of collecting data (GYTS-self administered questionnaires, Shandong-similar with our study, assisted by trained interviewers) [25, 26].

Our results show that parental migration is associated with the smoking behavior of children. The analysis makes clear that the rate of smoking of LBC in families in which the mother outmigrates (but the father is home) and in families in which both parents outmigrate is greater than in families in which children live with both parents. Furthermore, the age of LBC was statistically younger than non-LBC. The rate of smoking of LBC might be underestimated. The difference of the rate of smoking between the LBC and might be greater. More general studies indicate that a stable family environment is important for adolescents who are in their critical stage of development [12, 13, 18]. Living together with parents will improve parent-child communication, which may reduce children’s unhealthy behavior [12, 13]. As a result, it is not surprising that parental migration appears to play a significant role in LBC smoking behavior. Such a result implies that even though parental migration might have short-term financial benefits to a family, it may also have a negative effect on the health behavior of children.

Several studies have found that there is a gender difference in the rate at which children smoke [27, 28]. Similar with earlier studies, we observe a large gender disparity among the LBC in this study, with 8.2 % of boys and only 1.2 % of girls smoking. In China, there is a big gender difference in smoking behavior among adults. In our study province, a survey indicated that 67.0 % of men had smoking behavior, but only 2.6 % of women were current smokers [22]. This role model might be an important reason for the gender difference among adolescents found in our study. Another possible reason may be that cultural norms encourage young men to start smoking. It is often said that in rural China males should be more adventurous, and one way to demonstrate this is to engage in smoking; in contrast, there appear to be fewer cultural reasons for females to smoke [27–29]. In fact, in China parents and grandparents often believe that females should be modest and responsive to the authority of caregivers [28], which may also limit smoking among young females.

Previous studies have suggested that narrowing the gender gap in smoking behavior, especially in the case of adolescents, would be helpful for shrinking gender disparity in life expectancy [30]. However, there is little attention in the literature on developing sex-specific interventions for tobacco use control when targeting children. Lessons learned from studies on why the rate of smoking among females is lower may be useful for developing strategies on gender gap reduction in current smoking behavior among LBC.

We find that grandparents are most likely to be caregivers of LBC when both parents are absent, followed by other relatives. As expected, those with one migrant parent and one parent at home are cared for by the parent at home.. Differing from other studies [19, 20], however, we find that there is a significant association between the type of caregiver and LBC smoking behavior. This indicates that care from other relatives may have adverse effects on LBC smoking behavior. Compared with LBC that are cared for parents or grandparents, those who were cared for by other relatives may be more at risk for neglect and exploitation [25], which in turn may lead to increased tobacco use. Our study also found that academic performance was related with current smoking rate among LBC, those who perceived poorer academic performance tended to be higher frequent current smokers.

In China, anti-smoking campaigns had been issued to reduce the prevalence of smoking behavior among children, including policies on banning smoking in primary and secondary schools, forbidding to sell tobacco to minors. The findings from this study would be useful for developing tobacco control interventions among children. The results show that parental migration would increase the risk of current smoking behavior of children, which implicates a need to build a social support network targeting LBC from potential providers, including teachers, classmates and other caregivers, to offset the negative effect of parental migration on smoking behaviors of children. Our research also shows that perceived school performance is associated with smoking behavior among LBC. It is vital to develop school-based remedial classes targeting LBC who perceived poorer school performance. The intervention may not only lead to a reduction in smoking prevalence, but also a human capital investment for their futures. Interestingly, we found that those LBC cared by other relatives were more likely to be current smokers. This finding implies for those parents that when they decide to outmigrate, they should consider the capabilities of the caregivers who will replace them. If possible, they should not leave their children to other relatives for care. 

The study has several limitations. First, it is a cross-sectional study, and, therefore, can be only used to measure associations between smoking behavior and parental migration. The relationships that we identify cannot be interpreted as causal in nature. Second, the selection of LBC might be biased. The survey is conducted during the summer holiday time period. It might be that some LBC left the sample sites and were in the city with their parents during the period of the survey. Such children were not included in the study. This might affect the difference between LBC and non-LBC found in this study. Third, even though we have minimized the estimation error by helping the students in their recall efforts, the measure of socio-demographic and smoking behavior data were self-reported, meaning that a self-reporting bias may affect our results. Fourth, some important information were not collected in our study, such as smoking habits of primary caregivers, social integration, social support, parental attitudes to children’s smoking behavior and also the reasons for initiating tobacco use. It might affect the result of our study. Furthermore, there were only five current smokers among those whose “mother out, father home” and only four smokers among female LBC. To some extent, this small numbers might affect the result. In spite of these limitations, this study adds some new values. First, we find that LBC, especially those whose parents both migrated, tend to be current smokers. Second, different from previous studies, our research result indicates that the LBC who perceive poorer school performance are more likely to be current smokers.

## Conclusion

Our study suggests that parental migration is associated with increased risks of smoking by children. The study also finds a gender difference in smoking behavior among LBC. A sex specific intervention for the control of tobacco use may be needed. LBC perform poorer academically also tend to have higher rates of smoking. Given the significant costs associated with healthcare for tobacco users when they reach adulthood and beyond, some public health actions and anti-smoking intervention studies that target youth and in particular vulnerable youth are likely to be worthwhile.

## Abbreviations

GDP, gross domestic product; LBC, left-behind children
